# Context-dependent representation of within- and between-model uncertainty: aggregating probabilistic predictions in infectious disease epidemiology

**DOI:** 10.1098/rsif.2022.0659

**Published:** 2023-01-25

**Authors:** Emily Howerton, Michael C. Runge, Tiffany L. Bogich, Rebecca K. Borchering, Hidetoshi Inamine, Justin Lessler, Luke C. Mullany, William J. M. Probert, Claire P. Smith, Shaun Truelove, Cécile Viboud, Katriona Shea

**Affiliations:** ^1^ Department of Biology and Center for Infectious Disease Dynamics, The Pennsylvania State University, University Park, PA, USA; ^2^ Eastern Ecological Science Center at the Patuxent Research Refuge, U.S. Geological Survey, Laurel, MD, USA; ^3^ Department of Epidemiology and Carolina Population Center, Gillings School of Global Public Health, University of North Carolina at Chapel Hill, Chapel Hill, NC, USA; ^4^ Department of Epidemiology, Bloomberg School of Public Health, Johns Hopkins University, Baltimore, MD, USA; ^5^ Applied Physics Laboratory, Johns Hopkins University, Baltimore, MD, USA; ^6^ Department of International Health, Bloomberg School of Public Health, Johns Hopkins University, Baltimore, MD, USA; ^7^ Big Data Institute, Li Ka Shing Centre for Health Information and Discovery, University of Oxford, UK; ^8^ Fogarty International Center, National Institutes of Health, Bethesda, MD, USA

**Keywords:** combination, uncertainty, ensemble, multi-model scenario projections, linear opinion pool, Vincent average

## Abstract

Probabilistic predictions support public health planning and decision making, especially in infectious disease emergencies. Aggregating outputs from multiple models yields more robust predictions of outcomes and associated uncertainty. While the selection of an aggregation method can be guided by retrospective performance evaluations, this is not always possible. For example, if predictions are conditional on assumptions about how the future will unfold (e.g. possible interventions), these assumptions may never materialize, precluding any direct comparison between predictions and observations. Here, we summarize literature on aggregating probabilistic predictions, illustrate various methods for infectious disease predictions via simulation, and present a strategy for choosing an aggregation method when empirical validation cannot be used. We focus on the linear opinion pool (LOP) and Vincent average, common methods that make different assumptions about between-prediction uncertainty. We contend that assumptions of the aggregation method should align with a hypothesis about how uncertainty is expressed within and between predictions from different sources. The LOP assumes that between-prediction uncertainty is meaningful and should be retained, while the Vincent average assumes that between-prediction uncertainty is akin to sampling error and should not be preserved. We provide an R package for implementation. Given the rising importance of multi-model infectious disease hubs, our work provides useful guidance on aggregation and a deeper understanding of the benefits and risks of different approaches.

## 1. Introduction

Predictions about the future are central to describing and managing ecological systems. In these complex and uncertain settings, the decision-making process relies on identifying what could happen in the future and the likelihood of those potential outcomes [1,2]. The optimal policy decisions that use these predictions often depend strongly on the associated uncertainty. For example, a decision maker may be concerned with the risk of exceeding some threshold (e.g. estimating the chance a hospital will exceed bed capacity), or uncertainty may yield a qualitative change in the decision recommendation (e.g. implementing a mask mandate in response to a rise in projected COVID-19 transmission). As such, deliberate processes for appropriately expressing and managing uncertainty are warranted [3].

One such method involves eliciting, and then aggregating, predictions from multiple independent experts or models. In their seminal paper, Bates and Granger demonstrate the benefits of additional information provided by independent predictions, showing that an average of two predictions is more accurate than either alone [4]. Since then, aggregated predictions have been shown to more accurately and more reliably capture future outcomes than a single expert or model. Representing these predictions probabilistically (i.e. defining the probability of possible future events) provides the most complete expression of uncertainty and risk [5]. In the field of infectious diseases, efforts to elicit predictions across multiple mathematical and statistical models are becoming common to support planning and outbreak response [6,7]. Multi-model efforts have been used to predict a range of future public health outcomes (e.g. incident deaths, peak magnitude or epidemic size) [8–12] and estimate intervention effectiveness [13–15].

Importantly, the method used to aggregate multiple predictions has a meaningful, and often substantial, effect on the resulting ensemble. Despite the significance of aggregation methodology and an extensive available literature, identifying the most appropriate method for a given problem can be difficult. Furthermore, which method is most appropriate can depend on one's primary objective (e.g. some objectives proposed by Winkler [16] are overall performance, reliability and robustness to poor performance, and ease of communication and interpretation). Within multi-model infectious disease studies specifically, a range of aggregation methods have been adopted (e.g. averaging probability bins [9], averaging quantiles [12], performance-weighted average [17]). In this work, we review two classes of methods for aggregating multiple probabilistic predictions and illustrate their application in the context of infectious disease dynamics.

Validation of the performance of different aggregation methods against subsequent empirical data can inform methodological choices [18]. However, validation requires feedback between predictions and data that are impractical in many decision contexts. One particularly difficult case is that of scenario projections, namely when decision makers are interested in comparing predictions across multiple possible future situations [19]. In contrast to forecasts, which are predictions about the future (*what will happen*), scenario projections are conditional on a given set of assumptions about how the future will unfold (*what would happen if*); we use ‘prediction’ as a general term that encompasses both forecasts and projections. Scenarios may specify actions a decision maker could take in the future (e.g. implementation of, and assumed compliance with, a public health intervention) or uncertainties that are out of the decision maker's control (e.g. emergence of a new virus variant). Because scenarios are not expected to occur exactly as specified, it is unclear how best to compare projections with empirical data [20], complicating potential performance assessment of different aggregation methods.

Similarly, when the timeframe over which a decision must be made is short relative to the availability of data for validation, an aggregation method must be chosen before it is possible to evaluate performance. The relative scales of these timeframes may depend on the natural history of a particular pathogen (e.g. time to symptom presentation in influenza versus tuberculosis) or the decision context (e.g. hospital capacity planning versus setting eradication and elimination targets). Some resource allocation decisions, for example, require quantitative predictions that need to be made well in advance of anticipated need, yet by the time validation of those predictions is possible, the decision-making window has passed.

While eliciting predictions from multiple models provides useful information for infectious disease management, guidance is needed on how to aggregate predictions when there are little to no empirical data available. We address this question from two perspectives: (i) a literature review on the aggregation of probabilistic predictions, and (ii) a simulation analysis aggregating predictions from multiple infectious disease models in which we control the uncertainty within and between models. We end with recommendations for the choice of an aggregation method when empirical validation is lacking and illustrate how the chosen approach should be based on a hypothesis about the expression of uncertainty within and between individual model predictions.

## 2. Aggregation theory and methodologies

In this section, we summarize existing theory on two classes of methods to aggregate probabilistic predictions. We searched the literature from fields including expert judgement, statistics, operations research, climate modelling and forecasting of weather and economics. Our search terms included variations of ‘aggregation’, ‘combination’ or ‘ensemble’ across multiple databases such as Web of Science and ProQuest. We followed up on relevant articles to the extent possible through references therewithin. As the literature on these topics is vast, it is unlikely our search was exhaustive, but we pursued leads until converging on a set of similar concepts and references. As such, this section is not intended to be a formal, systematic literature review, but instead serves as an overview of concepts necessary to understand our case study.

In addition to summarizing relevant theory, we developed a package, CombineDistributions, in the R statistical software for implementing the described methods (https://github.com/eahowerton/CombineDistributions). This package supplements other existing packages, including for aggregating point estimates [21], aggregating and evaluating interval forecasts [22] and evaluating probabilistic predictions [23].

### 2.1. What constitutes a ‘good’ prediction?

**Figure 1.**
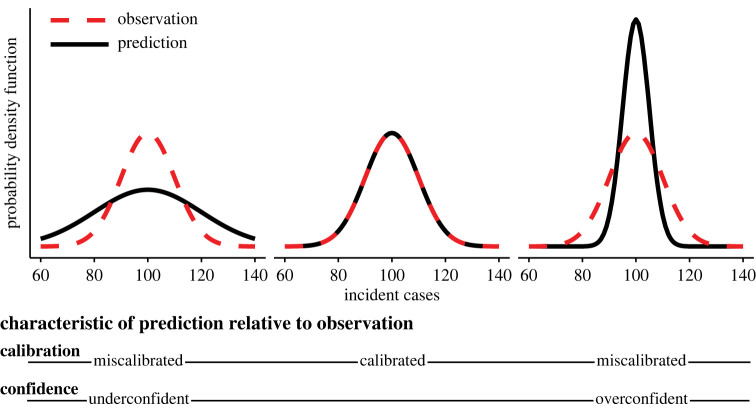
Black distributions represent three probabilistic predictions of incident cases and red dashed lines represent a distribution of ‘true’ observed incident cases (though in practice, we often expect to observe only one realization from this distribution). The calibration and confidence of each prediction is defined. While this figure focuses on prediction variance of unimodal distributions, miscalibration can arise in many ways (e.g. prediction mean).

The quality of a probabilistic prediction is often assessed based on calibration [24] (figure 1). *Calibration* describes the consistency between the prediction and the observed data, where better-calibrated predictions more closely reflect future observations. Gneiting & Katzfuss [25] define calibration as ‘statistical compatibility of probabilistic forecasts and observations; essentially, realizations should be indistinguishable from random draws from predictive distributions’. Predictions can be miscalibrated because of misaligned mean or variance (or, indeed, higher moments). *Confidence* describes the calibration of the variance specifically. Overconfident distributions are overly concentrated (i.e. predictions are too certain about a future outcome), while underconfident distributions are overly dispersed.

In many settings, however, only a single observation is realized, making it difficult to discern whether a distribution is well calibrated. As such, calibration is typically computed over a series of predictions (e.g. weekly forecasts of incident deaths), and in this case, the sharpness of a distribution is used to distinguish between predictions from different sources that all capture an observation. *Sharpness* describes the width of the predicted distribution without reference to future observations, where narrower distributions are sharper. Together these concepts constitute a standard for determining the quality of a prediction: ‘maximize sharpness, subject to calibration’ [24]. In other words, distributions should be as narrow as possible (maximize sharpness) without sacrificing the ability to capture future observations (subject to calibration). As our discussion of this topic is brief and the literature is vast, we direct readers to a selection of other papers and reviews for more information (see [24–36] for theoretical properties and corresponding metrics for evaluation and [20,37,38] for approaches to evaluation specifically for situations when observations are not readily available).

### 2.2. How to combine individual predictions?

There are many ways to use multiple sources of quantitative information, including selecting a single source or aggregating across sources. While there are many methods proposed in the literature to perform such aggregation, here, we describe two classes of methods for aggregating probabilistic predictions: methods that operate on probabilities, and methods that operate on quantiles. When operating with an arithmetic mean, these methods are called the linear opinion pool, or probability averaging, and the Vincent method, or quantile averaging (table 1). Both methods yield a valid probability distribution.

**Table 1.** Comparing across averaging techniques, ‘method’ gives the mathematical definition, ‘properties’ gives formulas for mean (*μ*) and variance (*σ*^2^), and ‘underlying premise’ provides a description of the theoretical basis. Throughout, *F*(*x*) is a cumulative distribution function (CDF) defined for values, *x*, *F*^−1^(*θ*) is a quantile function (inverse of the CDF) defined for quantiles *θ*, *N* is the number of predictions to be aggregated, and *w* is a weight for averaging. Subscripts _LOP_, _V_ or *_i_* indicate the linear opinion pool, Vincent average, or individual prediction, respectively.

	linear opinion pool (LOP; probability averaging)	Vincent average (quantile averaging)
method	calculate average cumulative probability *F*, at each value, *x*, or	calculate average value, *x*, at each quantile, *F*^−1^, or
	$F_{\mathrm{LOP}}(x)=\overset{N}{\underset{i=1}{\sum }}w_{i}F_{i}(x)$	$F_{V}^{-1}(\theta )=\overset{N}{\underset{i=1}{\sum }}w_{i}F_{i}^{-1}(\theta )$
properties	both methods yield distributions with the same mean, which is the average of individual distribution means	
	$\mu_{\mathrm{LOP}}=\overset{N}{\underset{i=1}{\sum }}w_{i}\mu_{i}$	$\mu_{V}=\overset{N}{\underset{i=1}{\sum }}w_{i}\mu_{i}$
	the variance of the Vincent average will always be less than or equal to the variance of the LOP	
	$\begin{array}{rl} & \sigma_{\mathrm{LOP}}^{2}=\;\overset{N}{\underset{i=1}{\sum }}w_{i}\sigma_{i}^{2}+\;\overset{N}{\underset{i=1}{\sum }}w_{i}(\mu_{i}-\mu_{\mathrm{LOP}}{)}^{2}\\ & =\left(\begin{array}{c} \mathrm{mean}\;\mathrm{of}\\ \mathrm{individual}\;\\ \mathrm{variances} \end{array}\right)+\left(\begin{array}{c} \mathrm{variance}\;\mathrm{of}\\ \mathrm{individual}\;\\ \mathrm{means} \end{array}\right) \end{array}$	although $\sigma_{V}^{2}$ follows no general form, $\sigma_{V}^{2}\leq \sigma_{\mathrm{LOP}}^{2}\;\mathrm{and}$ $\sigma_{V}^{2}\;=\;\overset{N}{\underset{i=1}{\sum }}w_{i}\sigma_{i}^{2}$ if all predictions are from the same location-scale family
underlying premise	each prediction captures a possible outcome, and therefore *between-prediction uncertainty is retained*	each prediction represents a noisy sample from a higher-order distribution, and therefore *between-prediction uncertainty is not retained*

**Figure 2.**
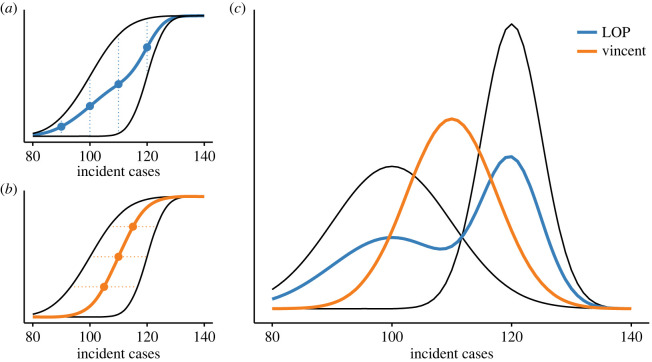
(*a*) The linear opinion pool (LOP; blue) averages *probabilities* across individual predictions (black) along a single vertical line. (*b*) The Vincent average (orange) averages *values* across individual predictions (black) along a single horizontal line. (*c*) Probability density functions for individual predictions (black) and each aggregation method (LOP, blue; Vincent average, orange). In all panels, black lines show individual predictions to be aggregated, *N*(*μ* = 100, *σ* = 10) and *N*(*μ* = 120, *σ* = 5). The Vincent average is equivalent to averaging individual distribution parameters, or *N*(*μ* = 110, *σ* = 7.5). Panels (*a*) and (*b*) are modified from [40] and panel (*c*) from [41].

The linear opinion pool (LOP) [39] is calculated by taking the arithmetic mean of cumulative probabilities (or, equivalently, probability densities) across alternative predictions for a fixed *x* value (averaging in the ‘vertical direction’, figure 2*a*). Alternatively, the Vincent average [42,43] is calculated by taking the arithmetic mean of values across alternative predictions for a fixed quantile (averaging in the ‘horizontal direction’, figure 2*b*). The median of the Vincent average will be the arithmetic mean of individual model medians. If the predictions to be aggregated are not defined analytically, implementing these methods numerically requires a means of interpolating the cumulative distribution function (CDF) between defined value-quantile pairs.

An array of existing theory describes these two methods, including cases in which each method is preferred. Although both methods produce an aggregate distribution with the same mean [40], there are many ways in which the resulting aggregates differ. The Vincent average will be sharper than the LOP applied to the same set of predictions [40], due to the increase in LOP variance ($\sigma_{\mathrm{LOP}}^{2}$, table 1) as individual predictions become less sharp or more dispersed in central tendency [44]. As individual prediction means are more dispersed, the two methods become increasingly dissimilar [41].

Importantly, theoretical results have shown that using LOP to aggregate a set of well-calibrated distributions will only be well calibrated if the individual CDFs are identical [45–47]. Methods that use metrics other than a weighted average have been proposed to compensate for the overdispersion of the LOP (e.g. logarithmic [41,48], beta-transformed [47], spread-adjusted [45] and generalized [29,49] linear pools). However, in cases where individual distributions are overconfident and somewhat dispersed in central tendency, the simple arithmetic mean LOP can yield a better calibrated distribution because of the increased variance [46].

Unlike the LOP, the Vincent average can preserve distributional shape across predictions for some distributions. Specifically, when all individual predictions are from the same location-scale family (i.e. distributions defined by only location and scale parameters, such as normal, logistic, Cauchy), the Vincent average will also be from that family and the parameters of the aggregate will be the average of individual distribution parameters [50].When the true distribution is from the same family as the individual distributions, this shape-preserving property can offer calibration benefits [40]. Other reviews provide further discussion of the theoretical properties of these methods [40,41,51].

Importantly, these properties emerge from assumptions underlying each method about how uncertainty is expressed between predictions. Here, and throughout, we use ‘between-prediction’ uncertainty to refer to the uncertainty captured across the set of independent predictions; this does not imply predictions are related in any way. The LOP treats individual predictions as alternative possible futures across which uncertainty should be retained [52], yielding an aggregate distribution that superimposes the shapes of each individual prediction. The Vincent average assumes individual predictions are each an imperfect representation of a single distribution of interest, appropriately capturing uncertainty despite random noise across predictions. As such, the Vincent average cancels high and low predictions, yielding an intermediate aggregate distribution. We demonstrate this philosophical difference by aggregating two normal distributions (figure 2*c*). The LOP is bi-modal with modes centred at each of the two individual distributions, whereas the Vincent average is centred between the two individual distributions.

### 2.3. How should predictions be weighted?

In addition to the averaging direction, the weights assigned to each prediction (i.e. *w_i_* in table 1) affect the resulting aggregate distribution. Weighting schemes can recalibrate the aggregate by giving more weight to individual predictions that perform well (e.g. [4,53,54]). Despite the prevalence of sophisticated optimization schemes (e.g. machine learning algorithms [18]), giving all predictions equal weight is surprisingly robust in many applications [55–57] including some infectious disease forecasting settings [54]. However, averaging can be sensitive to outlying predictions, and in the LOP framework, this sensitivity can lead to a highly dispersed aggregate distribution. We describe one method, called trimming, which can address this concern without the need for feedback with observations.

Trimming methods adjust the sharpness of the aggregate distribution by excluding some values (i.e. assigning a weight of zero) and equally weighting all remaining values [58]. Exterior trimming, which gives zero weight to outermost values, increases the sharpness of the aggregate distribution. This approach is expected to improve performance in cases where the unweighted aggregate is underconfident, including for example, aggregating predictions that vary greatly in central tendency [58]. Aggregating with a median instead of a mean is the most extreme form of exterior trimming, where all values except the centremost are given zero weight. The median LOP and median Vincent average are equivalent in most cases, and the reduced variance of the median LOP can lead to performance improvements in cases where the unweighted (mean) LOP is underconfident [59].

Interior trimming gives zero weight to central values, decreasing the sharpness of the resulting distribution by increasing weight on outer values. This trimming method is appropriate when individual predictions are overconfident and concentrated in central tendency [58]. The effect of trimming a single value depends on the number of predictions being aggregated (e.g. trimming one value in a set of five predictions excludes 20% of available information compared with 2% in a set of 50 predictions).

**Figure 3.**
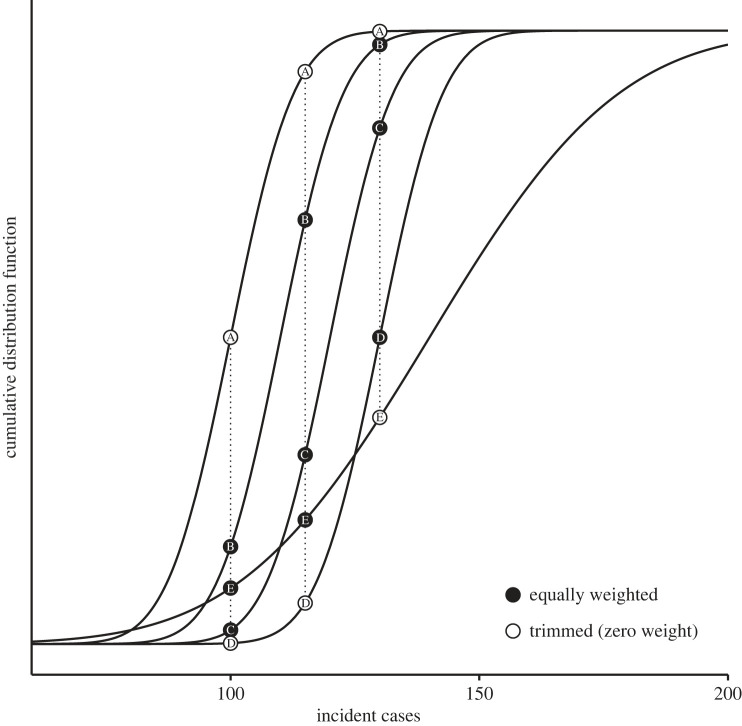
Solid black lines show individual predictions to be aggregated (A: *N*(*μ* = 100, *σ* = 10), B: *N*(*μ* = 110, *σ* = 10), C: *N*(*μ* = 120, *σ* = 10), D: *N*(*μ* = 130, *σ* = 10), E: *N*(*μ* = 140, *σ* = 30)). At three values of incident cases (100, 115, 130), circles show which individual predictions were trimmed (open circles) and which were included (closed circles) when aggregating. Figure modified from [58].

These trimming strategies depend on a method for determining which values are ‘interior’ or ‘exterior’ (i.e. ranking). Here, we focus on CDF trimming [58] which ranks cumulative probabilities at each value. Figure 3 illustrates exterior CDF trimming, where open circles indicate cumulative probabilities that will be given zero weight in the LOP average. The individual prediction being trimmed can vary across values; for example, the predictions being trimmed are different at 100 and 130 incident cases (A and D versus A and E; figure 3). As a result, CDF trimming often includes information across more individual predictions than alternative trimming methods (e.g. mean trimming, which excludes entire distributions from aggregation based on the mean, details in electronic supplementary material, S1.1). More discussion and examples of trimming are provided in [58,59].

## 3. Illustrating the choice of aggregation method in a simulated outbreak setting

While it is preferable to evaluate the performance of different aggregation methods based on past empirical data, it is not always possible, and results can be difficult to interpret. In these cases, theory (as outlined in the previous section) provides a useful ground on which to make methodological decisions.

However, the needs of the infectious disease setting may not be fully met by the guidance available in existing theory. Much theory on aggregating probability distributions depends on assumptions about the form of the underlying predictive distribution (e.g. normal distribution), but infectious disease predictions may not be of the studied forms or any particular form at all. Here we illustrate how the choice of aggregation approach can affect the resulting predictions, specifically for an infectious disease setting. We simulate the aggregation of predictions from multiple infectious disease models to predict two disease burden outcomes and investigate the properties of LOP- and Vincent-aggregated distributions. We focus on the equally weighted aggregation methods to illustrate the assumptions of each approach and then explore the properties of exterior trimming when an individual model with outlying assumptions is included in the set.

### 3.1. Methods

We use different versions of a simple SIRS (susceptible-infected-recovered-susceptible) epidemiological model [60]. In this model, susceptible individuals become infected based on the transmission rate, *β*. Infected individuals clear infection and move to the recovered class based on the recovery rate, *γ*. Once recovered, individuals are immune and cannot be reinfected for some time; the immunity of such recovered individuals, however, can wane at rate *ρ*, moving that individual back to the susceptible class. When *ρ* = 0, the SIRS model is equivalent to an SIR (susceptible-infected-recovered) model for fully immunizing infections (i.e. infections for which immunity does not wane).

Within our simulation, we define four individual models that make predictions about an outbreaking infectious disease in a population of 1000 individuals who are all susceptible at the start of the outbreak. Individual models make a single prediction of cumulative and peak cases that will occur in a 52-week period (i.e. the total number of new infections in that timeframe and the maximum number of new infections in a single week of that timeframe, respectively). Our simulations capture uncertainty about both model parameters (i.e. parametric uncertainty) and model structure (i.e. structural uncertainty) (table 2). We implement parametric uncertainty within and between models. For within-model parametric uncertainty, we let individual models draw the transmission rate from a normal distribution with some mean, *μ*_β_, and variance, $\sigma_{\beta }^{2}$. To implement between-model parametric uncertainty, we assume *μ*_β_ varies across models.

**Table 2.** Individual model parameters across three cases of uncertainty expression within and between models. Assumptions are shown for each model (A, B, C, D, E). For simplicity, we assume that all models define *σ*_β_ = 0.2 and 1/*γ* ∼ *N*(*μ* = 1, *σ* = 0.1). For the susceptible-infected-recovered (SIR) model, *ρ* = 0, for the susceptible-infected-recovered-susceptible (SIRS) model, we assume a 26-week mean time to waning (i.e. *ρ* = 1/26), and for models that represent both waning possibilities, SIR and SIRS, we assume *ρ* ∈ {0, 1/26}.

	model	parametric uncertainty		structural uncertainty	
case 1	A	within & between models	*μ*_β_ = 1.2	none	SIR
	B		*μ*_β_ = 1.4		SIR
	C		*μ*_β_ = 1.6		SIR
	D		*μ*_β_ = 1.8		SIR
	E		*μ*_β_ = 2.4		SIR
case 2	A	within & between models	*μ*_β_ = 1.2	between models	SIR
	B		*μ*_β_ = 1.4		SIRS
	C		*μ*_β_ = 1.6		SIR
	D		*μ*_β_ = 1.8		SIRS
	E		*μ*_β_ = 2.4		SIR
case 3	A	within & between models	*μ*_β_ = 1.2	within models	SIR & SIRS
	B		*μ*_β_ = 1.4		SIR & SIRS
	C		*μ*_β_ = 1.6		SIR & SIRS
	D		*μ*_β_ = 1.8		SIR & SIRS
	E		*μ*_β_ = 2.4		SIR & SIRS

We assume the primary source of structural uncertainty is about waning of immunity, specifically whether the infection process follows an SIR (*ρ* = 0) or SIRS (*ρ* greater than 0) model. To understand the impact of structural uncertainty on aggregation, we consider three cases about structural uncertainty:

(1) Structural uncertainty is *not represented* across individual models: this case may arise in situations where there is consensus on a particular biological or epidemiological feature. Alternatively, all models may improperly make the same assumption due to a shared ignorance of the system (i.e. unknown unknowns). Here, we represent this case with all models assuming no waning immunity.
(2) Structural uncertainty is represented *between models*: as there are multiple ways to represent complex biological processes with mathematical prediction models, this case is probably common in multi-model prediction settings. Here, we represent this case with two models assuming waning and two assuming no waning.
(3) Structural uncertainty is represented *within models*: this case may arise in situations where an uncertainty that is known to be important (but has not yet been resolved) is included by independent models. Here, we represent this case with all individual models assuming with equal probability there will be no waning or waning.

For real multi-model prediction efforts, where there are many uncertainties affecting dynamics, it is most likely that multiple cases will be represented for a single set of predictions.

Within each case, we perform 100 000 stochastic replicates for each model using the chain binomial model [61]. This stochastic simulation framework assumes that the number of individuals transitioning between compartments (e.g. S to I) is randomly drawn from a binomial distribution. The probability of transitioning is based on the rate of that transition (defined by *β*, *γ* and *ρ*). Our simulation uses weekly timesteps, which may obscure early stochastic fade-out dynamics (such dynamics are not the focus of this simulation study).

Then, we summarize the stochastic replicates into a CDF approximated by a set of 999 equally spaced quantiles. We aggregate the CDFs using both LOP and Vincent average (with an equal weighting scheme) and test the performance of each aggregate under different assumptions about the future. To generate ‘future observations’ against which to test our aggregates, we make assumptions about the ‘true’ values of each parameter.

We consider realized futures where each individual model has captured *μ*_β_ (four futures), and where the true *μ*_β_ is the mean of individual model *μ*_β_s (one future). We cross these realized futures with two future waning scenarios, where immunity either wanes or does not wane (corresponding precisely to model assumptions, i.e. no waning implies *ρ* = 0 and waning implies *ρ* = 1/26). For each of these 10 realized futures (5 transmission futures × 2 waning futures), we generate 1000 synthetic observations against which we measure the performance of each aggregate distribution using the continuous rank probability score (CRPS) [62].

Last, we examine an alternative set of scenarios where an outlier model is included in the set of individual models to assess the utility of trimming. Outliers bias aggregate predictions, especially when there are only a handful of solicited models. But exterior trimming should, in theory, alleviate such biases. To simulate the effects of the outliers on aggregate distributions in an outbreak setting, we included an outlier model with *μ_β_* = 2.4, deliberately outside of the range of individual model and truth scenario values. We address the same three structural uncertainty scenarios: the outlier model assumes no waning immunity when structural uncertainty is not represented or represented between models; and assumes waning and no waning are equally likely when structural uncertainty is represented within models. We again aggregate these (now five) distributions using both LOP and Vincent average. We contrast equal weighting of both the LOP and Vincent average with CDF exterior trimming (excluding the highest and lowest values). We compare the performance of these four aggregate distributions in the 10 truth scenarios (defined in the previous paragraph) using CRPS.

All technical details for this simulation experiment, including model structure, output aggregation, evaluation and outlier case are provided in electronic supplementary material, S2. Code to implement this case study can be found in the SIRS vignette of the CombineDistributions R package.

### 3.2. Results

**Figure 4.**
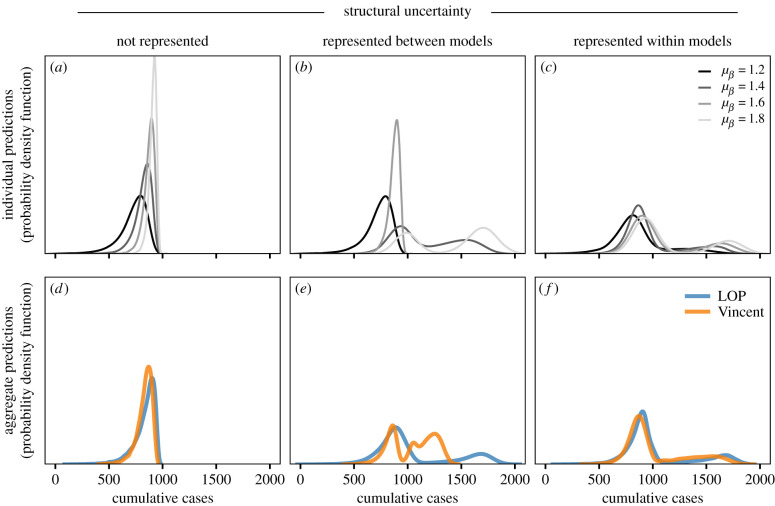
(*a*–*c*) Individual model predictions of cumulative cases over a 52-week period, for three assumptions about how structural uncertainty is represented across models. Each curve shows the distribution of cumulative cases generated from 100 000 stochastic replicates for four models that make different assumptions about transmission rate (grey colours, models A–D in table 2). (*d*–*f*) Aggregate distributions are shown when each set of four predictions are aggregated using equally weighted linear opinion pool, LOP (blue) and Vincent average (orange).

Our multi-model simulation illustrates the effect of uncertainty on individual model predictions and the corresponding aggregate under various methods. Predictions of cumulative cases depend on both parametric and structural assumptions of an individual model (figure 4*a–c*). For all models, cumulative case predictions increase with transmission rate (and corresponding *R*_0_). When a model assumes there is no waning, the population size (1000 individuals) serves as an upper bound on cumulative cases and predictions are left-skewed. Alternatively, models that assume immunity wanes yield multi-modal predictions with alternative states corresponding to stochastic fade-out after the first wave (500–1000 cumulative cases) and endemicity (1500–2000 cumulative cases). In very few cases, simulated epidemics fade out before an outbreak can take off (because our simulation is implemented in weekly timesteps). Although also yielding a multi-modal prediction, models that consider both waning possibilities assign different probabilities to each mode compared with those assuming immunity strictly wanes.

Differences in individual predictions translate into different aggregate distributions across the three structural uncertainty scenarios. In cases where assumptions about model structure are consistent across individual models, the LOP and Vincent average generate similar distributions (figure 4*d,f*). However, when individual models make different assumptions about model structure, the aggregate distributions are qualitatively different (figure 4*e*). In this case, although both methods generate multi-modal distributions with one mode at 500–1000 cumulative cases, the second mode in the LOP distribution is between 1500 and 2000 cumulative cases compared with 1000 and 1500 cumulative cases in the Vincent distribution. This second mode of the Vincent average leads to poor performance against possible future observations. Specifically, when compared against 10 possible truth scenarios varying the true mean transmission rate and waning immunity, the LOP (generated when individual models make different assumptions about structural uncertainty) has better CRPS values than the Vincent average for 84% of simulated observations (see §3.1 and electronic supplementary material, S2.2 for additional details).

**Figure 5.**
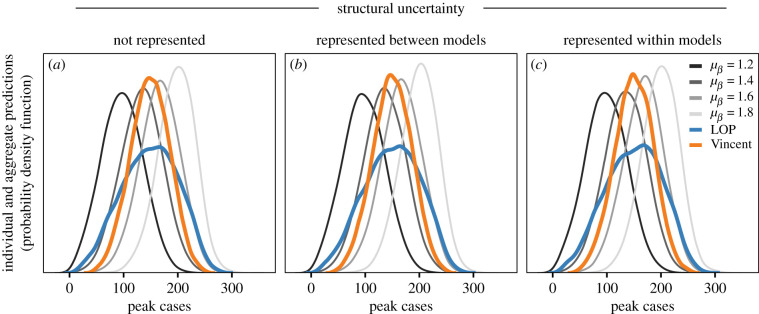
(*a–c*) Individual model predictions of peak cases over a 52-week period, for three assumptions about how structural uncertainty is represented across models. Each curve shows the distribution of peak cases generated from 100 000 stochastic replicates for four models that make different assumptions about transmission rate (grey colours, models A–D in table 2). Aggregate distributions when each set of four predictions are aggregated using equally weighted­­ linear opinion pool, LOP (blue) and Vincent average (orange).

Predictions of peak cases are less sensitive to structural uncertainty (as subsequent infection waves due to waning are smaller than the initial outbreak). As a result, parametric uncertainty is the major driver of differences between individual model predictions of peak cases (figure 5*a–c*). Both LOP and Vincent averages yield distributions with similar central tendency; however, the Vincent average retains the shape of the individual distributions and is sharper than the LOP, resulting in better CRPS values than LOP in 54% of the future observations considered. When the true transmission rate is the mean of individual model transmission rates (i.e. the case where individual model variation is akin to sampling error), the Vincent average performs better for 71% of simulated observations (electronic supplementary material, S2.2).

**Figure 6.**
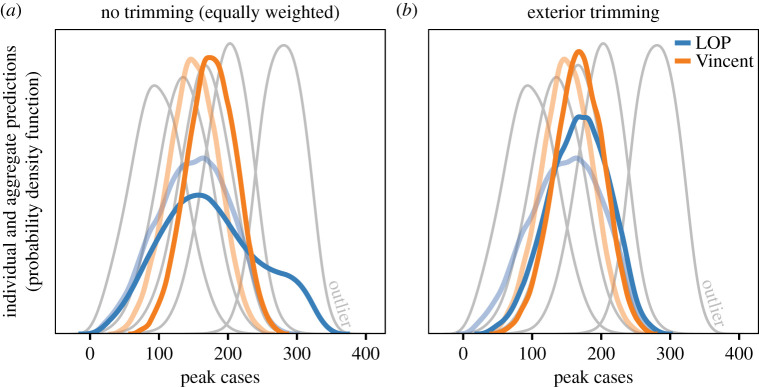
Two versions of LOP (blue) and Vincent average (orange) aggregate distributions for peak cases when an outlier is included in the set of individual predictions; (*a*) equally weighted (i.e. no trimming) and (*b*) exterior trimmed. For reference, the five models being aggregated are shown in grey, including the outlier (with *μ_β_* = 2.4, model E in table 2) which is labelled as ‘outlier’. The other four models match those in figure 5 (*μ*_β_ = 1.2, 1.4, 1.6, 1.8, models A–D in table 2). The LOP and Vincent average aggregate distributions without an outlier present are shown in lighter blue and orange, respectively. Results are shown for only the case where structural uncertainty is represented between models, as the three structural uncertainty scenarios have very similar predictions of peak cases (see electronic supplementary material, S2.3).

The presence of an outlier in the set of individual model predictions can affect the aggregate distribution and its performance when all models are weighted equally (our base scenario). Using peak cases as an example, the central tendency of the LOP and Vincent average aggregate distributions are shifted toward the outlier. While the sharpness of the Vincent average is relatively unaffected, the LOP becomes more dispersed with an outlier in the set (figure 6*a*). Exterior trimming reduces the effect of the outlier, slightly compensating for central tendency and sharpening the resulting LOP aggregate, compared with the untrimmed LOP (figure 6*b*). The trimmed LOP performs better than the untrimmed LOP for 69% of future observations considered; however, the Vincent average has the best performance across the majority of future observations (30% trimmed and 29% untrimmed). Similar results hold for predictions of cumulative cases, but here, a version of the LOP aggregate performs best for 70% of observations (44% trimmed and 26% untrimmed). See electronic supplementary material, S2.3 for detailed performance results, including the scenarios in which each method performs best, the magnitude of this performance advantage, and the variability of performance over multiple replicates.

## 4. Discussion

To be most useful for decision making and planning, predictions about the future (or possible futures) need to appropriately capture our current understanding of uncertainty, so the decision makers can assess risk. Representing predictions probabilistically is important for quantifying uncertainty, and aggregating predictions across multiple independent sources can help us more accurately express the uncertainty and incumbent risk.

When aggregating, methodological choices can significantly affect the way uncertainty is expressed. Identifying which aggregation method provides the most appropriate expression of uncertainty is a key challenge, especially in the absence of feedback with observations. When empirical validation is not possible (either not on relevant time scales or not at all, e.g. for scenarios that never materialize), we argue the chosen aggregation approach should align methodological theory with a hypothesis about the uncertainty represented within and between individual model predictions.

In particular, based on our review, theory suggests that when the uncertainty expressed between individual predictions should be retained, the properties and assumptions of LOP are more appropriate. However, when the between-prediction uncertainty is akin to sampling error and therefore should be averaged away, the Vincent average is better suited and the LOP will be underconfident. Beyond the direction of averaging, weighting schemes provide an additional means to calibrate the aggregate and modulate its variance. In cases where individual predictions vary in levels of confidence (with some probably underconfident) or are overly dispersed in central tendency, exterior trimming (including aggregating with a median) will reduce the variance of the aggregate. Identifying an effective weighting scheme is best achieved when predictions can be directly confronted with observations, yet simple rules may still be preferred [16].

There are challenges to identifying which theoretical case is more appropriate. Details about the assumptions and implementation of individual models may not be available, inhibiting identification of individual and collective expressions of uncertainty. Multiple uncertainties represented within the same set of predictions may need to be treated differently, or predictions may group into subsets based on similar assumptions, requiring one approach within a subset and a different approach across subsets.

Crucially, the choice of aggregation method can have a meaningful impact on decision making. Inappropriately aggregating with LOP will overestimate the probability of extreme events and may suggest more drastic actions than necessary. Conversely, inappropriately using the Vincent average may underestimate these probabilities, leaving decision makers unprepared. Identifying qualitative and quantitative decision outcomes across a range of aggregation methods will reduce the importance of selecting a single best method [52].

To further explore these conclusions in the context of infectious diseases, we used an outbreak simulation case study, where we defined exactly how uncertainty was expressed within each individual model and across the set. When individual models made different assumptions about waning immunity, the LOP retained important between-prediction uncertainty (namely about model structure) for predictions of cumulative cases. The Vincent average generated an aggregate distribution with a mode between the two expected alternative states (stochastic fade-out or endemicity).

A common criticism of the LOP approach is that it can be underconfident and sensitive to outliers. Here we have shown that a simple exterior trimming procedure can appreciably sharpen the aggregate LOP distribution and restore central tendency when outliers are present, with the trimmed LOP outperforming the untrimmed LOP for the majority of future observations considered. Here, outlier predictions were due to outlying model assumptions about the mean transmission rate, but there are a range of reasons outlier predictions may be generated (e.g. anomalies in data used to generate predictions could cause erroneous predictions). The likelihood of outlying predictions, and the reason these predictions are outliers (i.e. genuine scientific uncertainty versus technical error), may influence the chosen weighting scheme for aggregation.

The effect of each source of uncertainty was not consistent across prediction targets. For predictions about peak cases, variation across individual models was driven primarily by parametric uncertainty rather than structural uncertainty. Predictions of peak cases were not affected by waning immunity (i.e. structural uncertainty) because the largest peak in the SIRS model occurs during the first epidemic wave, before waning takes place. The first peak in the SIRS model is similar to the sole wave of the SIR model. This conclusion, however, may be context dependent as the peak in both models is sensitive to assumptions about initial conditions and parameters and becomes increasingly complicated as transmission rates and waning rates vary in time (e.g. as seen in the COVID-19 pandemic). If we consider individual model assumptions about transmission rate to be variation around a true mean (as we did in two of the ten truth scenarios considered), the sharper Vincent average provides a better representation of uncertainty for predictions of peak cases, including preserving the consistent shape of the individual predictions.

Here, we have presented a simulation study where the models generating individual predictions and future observations are known by design. Yet many additional challenges exist when implementing multi-model aggregation methods in practice. First, our simulated results necessarily are consistent in how outcomes are defined. However, achieving consistency across multiple independent models is not straightforward and failing to do so can lead to discrepancies in results [13]. Expert judgement methods can help to minimize the linguistic uncertainty associated with interpretation differences [3], and when consistency across models is not possible, vote-processing methods can be used to combine decision recommendations from differing sources [63]. In addition, we have assumed that observations were generated by parameters and processes within the range defined by the individual models, which is unlikely to be the case (e.g. truth values were simulated from the same general model structure and stochastic simulation framework as the individual models that generate the aggregate distribution). Identifying failures to capture observations among all models, including the aggregate, is an important first step to detecting and addressing changes in system dynamics that affect predictions [64].

Accurate estimates of future outcomes and related uncertainty is important for effective infectious disease management, including the integration of formal decision theory into infectious disease applications [65–68]. Aggregating predictions from multiple experts or models has proven to yield better calibrated estimates of future outcomes both for infectious disease dynamics (e.g. [9,17,69–71]) and other fields (e.g. [55]). These methods are becoming increasingly common in infectious disease management; however, many outstanding challenges exist to maximize the utility of these approaches [6,7]. Here we address one of these challenges: in some crucially important decision settings, traditional approaches to selecting an aggregation method (namely, empirical validation) are not feasible on decision-relevant timescales. By providing a theory-based guide to aggregation methodology and extending this theory to infectious disease modelling via simulations, our work provides much needed support of the use of multi-model approaches in public health planning and response.

## Data Availability

The R package to implement methods discussed in this paper can be found in the public repository https://github.com/eahowerton/CombineDistributions, https://doi.org/10.5281/zenodo.7437280. Code for the case study is available in the SIRS vignette. Methodological details and additional results are provided in the electronic supplementary material [72].
